# Who regulates? Effects of scaffolding in system- and self-regulated learning

**DOI:** 10.3389/fpsyg.2025.1543024

**Published:** 2025-05-21

**Authors:** Aileen Herold, Tina Seufert

**Affiliations:** Department Learning and Instruction, Institute of Psychology and Education, Faculty of Engineering, Computer Science and Psychology, University of Ulm, Ulm, Germany

**Keywords:** learning systems, self-regulation, scaffolding, learning performance, self-efficacy, prior knowledge

## Abstract

**Background:**

In adaptive learning settings, fine-grained dynamic measurements of learner characteristics (e.g., prior knowledge) and system-based decisions (e.g., adjusting task difficulty) enable learners to follow an individual learning path. Thus, the system takes over the regulation of the learning process. According to self-regulated learning, the learners could also do this themselves. Scaffolding offers a possibility to support the learner in system-based settings and self-regulated learning situations by making system decisions comprehensible and compensating for problems in self-regulation.

**Objectives:**

This study aims to investigate the influence of the agency of regulation (self-regulation/system-regulation) in combination with scaffolding (with/without) on learning performance and self-efficacy.

**Methods:**

We conducted a 2 × 2 experimental study with *N* = 102 participants (students in psychology and teacher education), studying in a digital learning environment. The effect of agency of regulation was examined as an open research question. We hypothesized that learning performance and self-efficacy are higher in the groups with scaffolding. Metacognitive competencies and prior knowledge are analyzed as moderators. Data analysis was conducted using ANCOVAs and moderation analyses.

**Results:**

The results showed a significant main effect of scaffolding on learning performance (recall) and self-efficacy. Furthermore, the results revealed a significant moderation effect regarding prior knowledge. An interaction effect and a main effect for the agency of regulation were not found.

**Conclusion:**

In further studies, the role of scaffolding within the interplay of system-based learning and self-regulation should be investigated in more depth by including the design of scaffolds and individual self-regulatory processes.

## 1 Introduction

Adaptive learning approaches offer the possibility to take into account individual differences between learners (Shute and Zapata-Rivera, 2012; Tetzlaff et al., 2021). Learners differ, for example, in their cognitive, metacognitive, motivational, and affective prerequisites (Vandewaetere et al., 2011). Based on the constant measurement (e.g., through learning analytics) of the current states regarding these properties, system-based adaptations can support the individual learning process. One way to address the needs of the learner is to offer personalized learning paths that include didactically prepared learning materials (Shute and Zapata-Rivera, 2012). In doing so, systems can make adaptations in different ways, for example, by taking into account individual prior knowledge, current motivation, and learner preferences (Guo, 2022; Tetzlaff et al., 2021). There are controversial views on whether such regulation by the system is conducive to learning (Kerres and Buntins, 2020; Popenici and Kerr, 2017; Vandewaetere et al., 2011). The agency that is in control of the learning process repeatedly makes decisions on how to proceed with the learning process. In a system-driven learning path, for example, the system would decide which learning content or learning goal to focus on next. In contrast, in a self-regulated learning setting, the learner makes decisions. By monitoring the learning process, the learner can regulate and, for example, select learning content or adapt the strategy or goal according to the current situation. The agency of this regulation can thus lie either with the system or the learner (Shute and Zapata-Rivera, 2012).

Numerous empirical studies indicate that self-regulation is a key component of successful learning (Jansen et al., 2019; Panadero, 2017; Zheng, 2016), which raises the question of what impact external regulation by the system has on learning and whether learners should take over the regulation of their learning process themselves.

However, previous research shows that learners often have problems regulating their learning spontaneously and do not use regulatory strategies, even though they know them (Bannert, 2009; Lim et al., 2023). Simultaneously, system regulation may not be the best option for every learner, as decision-making processes may remain intransparent or learners may otherwise no longer be able to self-regulate their learning (Kalyuga, 2009; Kerres and Buntins, 2020; Vandewaetere et al., 2011). Irrespective of whether the learner or the system is responsible for the learning process (e.g., deciding on the next steps), problems can arise.

To address this gap, scaffolding as targeted assistance can help with both self-regulation and system-regulated learning by compensating for self-regulation deficits (Bannert, 2009; Guo, 2022; Lim et al., 2023) and making system decisions comprehensible (Khosravi et al., 2020).

### 1.1 The agency of regulation while learning

*System-regulation*. Adaptive systems enable new approaches to individualize the learning process. By monitoring the learners’ cognitive or motivational states and controlling their learning with data-driven adaptations, individual learning performance can be increased (Tetzlaff et al., 2021). The system takes responsibility for regulative processes of learning (e.g., adjustment of goals), thus learners cannot or do not have to regulate themselves. The extent to which successful learning processes are inhibited or supported by this is controversial. Vandewaetere et al. (2011) assume that regulation by the system can help compensate for self-regulation problems and thus positively influence learning outcomes. Harati et al. (2020) even argue that learners expect the system to take over regulative tasks (e.g., goal setting) for them. In their work they developed a theoretical model to conceptualize self-regulation in adaptive learning and designed a questionnaire to assess these skills. Their findings highlight the role of adaptive systems in facilitating self-regulated learning rather than simply replacing it. Kerres and Buntins (2020), in contrast, emphasize in their systematic literature review on recommender systems the risk that learners’ experience of autonomy and ability to self-regulate is impaired because system decisions are made in a kind of “black box,” and therefore they are not comprehensible to the learners. The instructional intention behind a decision thus remains hidden from the learners and brings them into a dependency on the system. Kalyuga (2009) also raises concerns about whether regulation by the system can sustain the development of self-regulatory competencies. A contrary assumption is that learners with low self-regulatory competencies rely on external support when learning in systems-based learning environments (Guo, 2022) and cannot solve complex tasks independently (Harati et al., 2020). The arguments for and against system regulation prove challenging and demonstrate that the system cannot simply replace learner self-regulation.

*Self-regulation*. Self-regulated learning (SRL) is a self-directed process in which the learner takes responsibility for planning, monitoring, regulating, and evaluating his or her learning (Zimmerman, 2008). Based on Zimmerman (2008), self-regulation comprises a cyclical process with three phases: Pre-thinking involves analyzing the learning task by setting goals and planning the course of action. The learner’s motivational beliefs (e.g., self-efficacy, intrinsic motivation, goal orientation) are also of importance in this phase. The learners ask themselves whether they will be able to handle the learning task at hand. During the performance phase, there are also volitional processes involved. The learner controls (e.g., attention focusing and task strategies) and monitors himself or herself. This involves a continuous comparison between the set goals and the current success during learning. In the reflection phase, self-related evaluations and reactions take place, such as causal attribution. Self-regulation therefore encompasses the control of cognitive, motivational, emotional, and metacognitive learning dimensions (Panadero, 2017).

Overall, we can see that learners who learn in a self-regulated way, have distinctive metacognitive competencies (e.g., regulation skills and awareness of their cognitive processes; Guo, 2022), and can process learning content deeper, enabling them to better solve complex tasks (Bannert et al., 2009). Yet, there is consistent evidence that many learners often struggle (e.g., production and usage deficits) to apply these strategies in a situational and self-regulatory manner. As a result, there is a large body of research promoting self-regulation (Bannert, 2009; Veenman et al., 2006).

Previous research has investigated both the promotion and the effect of self-regulation. A range of studies suggest that targeted self-regulatory interventions have a positive impact on learning outcomes (Jansen et al., 2019). The effect of self-regulation is often examined concerning self-regulatory interventions in the context of training (Sitzmann and Ely, 2011) or specifically self-regulation-promoting learning settings such as digital learning environments or freely selectable learning paths (Azevedo et al., 2005; Müller and Seufert, 2018; Schwonke et al., 2006). For example, several studies also compared self-paced and system-paced learning in terms of learning time and organization of information (Roy, 2004; Schmidt-Weigand et al., 2010). The balance between external and self-regulated learning has already been investigated in different learning settings. Some studies highlight the potential added value (e.g., better strategy application) of external regulation (Azevedo et al., 2008; Guo, 2022; Harati et al., 2020). Azevedo et al. (2008), for example, reinforce that their pretest-posttest design and think-aloud protocols provided empirical evidence on the benefits of external facilitation. At the same time, several studies raised concerns about whether external control limits self-regulation, autonomy, and learner motivation (Kerres and Buntins, 2020; Shirk, 2020). Thus, in addition to learning performance motivational factors should also be considered.

How much control learners have in a learning situation also influences motivational processes (Moos and Azevedo, 2008; Vandewaetere and Clarebout, 2011). In general, motivation is the intentional effort to achieve a specific goal. Learners are more likely to engage in this effort when they feel autonomous, competent, and socially connected (Deci and Ryan, 2008). Research on motivation in learning and performance contexts is extensive (Howard et al., 2021; Lazowski and Hulleman, 2016). Especially in learning environments where the learner has to control the sequence of learning content, the inclusion of motivational constructs such as self-efficacy is considered essential (Moos and Azevedo, 2008). Self-efficacy represents the learner’s assumption that he or she can successfully cope with the challenges of an activity (Bandura, 1986) and is either an individual personality trait or situation-specific in relation to the accomplishment of a task. It can be assumed that the learner’s self-efficacy also changes depending on the agency of regulation. One perspective is, that self-regulation can increase self-efficacy through the experience of competence by enabling learners to make appropriate learning decisions for themselves (Vandewaetere and Clarebout, 2011). In contrast, the system can strengthen self-efficacy by making individual adjustments so that learners can master the task with the abilities they currently have (Hurley, 2006; Wang et al., 2023; Zhang, 2022).

Overall, it can be stated that the question of the agency of regulation should take metacognitive, cognitive, and motivational components into consideration. Regardless of whether the responsibility for regulation lies with the system or with the learner, barriers can still arise in the learning process. Scaffolding offers the possibility to counteract these discrepancies.

### 1.2 Scaffolding as a supporting technique

Scaffolding is a concept from educational psychology that gained attention in research as early as the mid-1970s (Berthold et al., 2007) and is rooted in Vygotskys theories (Vygotskiĭ, 1978). In general, scaffolds are assistance such as prompts, hints, or worked examples that the learner receives from an external source (e.g., a teacher or intelligent tutoring system; Zheng, 2016). Continuous observation and measurement of learning behavior is required to provide support as needed. Beyond that, scaffolding can be direct or indirect, fixed/hard or adaptive/soft, and domain-specific or cross-domain support (Zheng, 2016). However, the definitions and distinctions of scaffolding vary in individual studies. Thus, aids can be shown to the learner more or less implicitly in the background (in the case of explicit or direct support, the learner would have to indicate that a hint was read), individualized (e.g., address specific needs of the learner) or refer to the learning process in general. In addition, scaffolding, in our case, goes beyond feedback by providing learners with perspectives about their further learning procedure (Zheng, 2016). Self-regulatory scaffolds are domain-independent supports that, ideally, address all phases of self-regulation from planning to reflection. Help can be provided to the learner via strategic, conceptual, procedural, or metacognitive procedures (Belland, 2017; Zheng, 2016). Although the function of scaffolding extends beyond assisting with self-regulation (e.g., motivational scaffolding), most research focuses on metacognitive or self-regulatory scaffolding to stimulate self-regulation. In this context, scaffolding has established itself as a promising approach for successful learning (Berthold et al., 2007; Lim et al., 2023; Molenaar et al., 2011).

*Effects of Scaffolding*. Previous empirical findings support that scaffolds can improve learning performance in diverse learning environments (Azevedo et al., 2022; L. Guo, 2022). Some of these studies differentiate the learning outcome according to Bloom’s hierarchical taxonomy (1956) of cognitive learning goals. Accordingly, the knowledge levels that built on each other range from recall (retrieval of knowledge), to comprehension (linking of knowledge), to application (transfer to other contexts). There are indications that supportive aids such as prompts have a learning-promoting effect, particularly in the case of transfer tasks. From a motivational perspective, scaffolding contributes to improved self-efficacy (Girasoli and Hannafin, 2008; Guo et al., 2023; Valencia-Vallejo et al., 2018). These findings are provided, for example, by studies in which prompts were used to promote self-efficacy (Gentner and Seufert, 2020; Müller and Seufert, 2018).

*Interaction of Scaffolding and Regulation*. In the context of technology-based learning paths, the sequence of steps can be adopted both by the learner and by the learning system (e.g., start learning with general definitions and then move on to practical tasks). Depending on who regulates this decision process, scaffolding can have different functions as supporting measures.

The situational measurement of the learning state, like the current knowledge level, enables the system to make decisions regarding the next steps for the learner. In the case of system-regulated learning, scaffolding thereby offers the opportunity to make hidden system processes (e.g., the selection of an appropriate task) comprehensible to the learner. The system can make adjustments in two ways: covertly or by sharing the information via scaffolds with the learner. Scaffolding then serves as a tool to reveal system decisions and thus make external regulation transparent to the learner, which may lead to a better understanding of the individual learning (Kerres and Buntins, 2020). Thus, Scaffolding can be conducive to successful learning in the case of a system-driven learning path.

In self-regulated learning situations, learners plan their next steps and set goals for their learning, e.g., which chapter to look at next (Veenman et al., 2006). Providing scaffolding for the learning process can serve as a metacognitive aid that helps learners to decide in a self-regulated manner. Without the appropriate help, they might not even know about the metacognitive action or might not perform it spontaneously. Learning performance can be increased by such situational scaffolding (Zheng, 2016).

If we consider the effect of the agency of regulation (self-regulation/system regulation) and scaffolding on learning performance, it becomes apparent that individual differences in learners’ metacognitive competencies and prior knowledge may also be relevant (Azevedo et al., 2005). If the system regulates, the effect of scaffolding on learning performance may depend on the level of metacognitive competencies. In a system-regulated setting learners with high metacognitive competencies might feel restricted regarding their decisions and degree of control. In this case, support such as scaffolding would probably be more disruptive to these learners because they do not need the extra help (Clarebout et al., 2010; Jansen et al., 2019). Learners with low metacognitive competencies, on the contrary, could be unburdened by the system and its decisions (Harati et al., 2020). The scaffolds could assist them in comprehending how the system acted (Kerres and Buntins, 2020). Considering the effect of scaffolding in self-regulated learning settings, it also makes a difference in learning performance whether the learners have sophisticated metacognitive competencies. In a self-regulated learning environment, learners who lack metacognitive competencies would probably feel more overwhelmed and thus would have a disadvantage in learning (Greene et al., 2011). Scaffolding would help them to compensate for this deficit (Bannert, 2009). Learners with extensive metacognitive resources could benefit from high control in this setting. Scaffolding could be taken advantage of for further reflection or metacognitive actions in that case (Zheng, 2016). It is therefore possible that a Matthew effect will occur. Learners with a great amount of metacognitive competencies benefit from the help, whereas learners with a low level of metacognitive competencies are at a disadvantage (Vandewaetere and Clarebout, 2011; Walberg and Tsai, 1983).

Furthermore, learners consistently vary widely in terms of prior knowledge, which should be accounted for in the interplay between the agency of regulation and scaffolding concerning learning. In system-regulated contexts, experienced learners with high prior knowledge could use scaffolding to comprehend the system’s decisions and gain an advantage in their learning (Vandewaetere and Clarebout, 2011). Irrespective of who regulates, learners without prior knowledge benefit more from instructional support such as scaffolding than more experienced learners, as learners with high prior knowledge already have internally developed mental models, which then overlap with the external instructions from the instructional aids (Kalyuga et al., 2003; Roelle and Berthold, 2013). We can therefore assume that novices in system-regulated learning situations would benefit from support regarding their learning, as they are supported by external instructions. This is why learners with low prior knowledge would benefit likewise from scaffolding in self-regulated learning environments. In contrast, individuals who have high prior knowledge are generally better able to navigate and cope effectively in self-regulated situations (Greene et al., 2010). They could utilize the control to their advantage, resulting in higher learning performance (Hannafin, 1984). But in combination with scaffolding, learners with high prior knowledge may find such support distracting, when having the possibility to regulate the learning themselves (Roelle and Berthold, 2013).

Therefore, this research aims to investigate how system regulation and self-regulation in combination with scaffolding affect learning performance and self-efficacy. Additionally, metacognitive competencies and prior knowledge of the learners are taken into consideration. Thus, this study aims to contribute to research on self-regulation in system-based learning environments and to extend knowledge about the diverse applications of scaffolding.

Table 1 provides an overview and examples of the study design.

**Table 1:** Examples of learning decisions depending on agency and scaffolding.

	No scaffolding = learner gets no supporting hint	Scaffolding = learner gets a supporting hint
Self-regulation = Learner makes decisions on the next steps in the learning process	The learner gets the feedback, if the test was passed and no hint where to go next. The learner decides which content to study next.	The learner receives the message ‘Well done! Based on your results, we recommend that you now look at the chapter on learning strategies’ and is free to go there or decide to take another step. If the test is not passed, the learner is advised to look at the same learning material again.
System regulation = System makes decisions on the next steps in the learning process	The learner gets the feedback, if the test was passed and the system leads him directly to the next learning content.	The learner receives the message ‘Well done! Based on your results, we recommend that you now look at the chapter on learning strategies’ and the system leads him to the next learning content. If the test is not passed, the learner is advised to look at the same learning material again.

### 1.3 Present study and hypothesis

From previous research, it is concluded that both the agency of regulation and scaffolding are influential in learning. To extend the findings on instructional aids in the context of self- and system-regulated learning in a practical context, we implemented this study as follows. In a digital learning setting, participants in a university course received either a system-based learning path with or without scaffolding or a self-directed learning path with or without scaffolding. During learning, learners completed continuous learning outcome measures that provided the basis for further action or decision-making as well as measuring learning performance as the dependent variable. Before the learning, learners also completed self-report questionnaires to assess prior knowledge and metacognitive competencies. After the learning unit self-efficacy was assessed.

Against this background, we investigate the research question, whether agency of regulation (system regulation/self-regulation) influences learning performance (RQ1a) and self-efficacy (RQ1b). We investigate the effects as an open research question (RQ 1), as previous findings and theories provide ambivalent arguments for each form of regulation.

As a second research question we investigate the influence of scaffolding on learning performance (RQ2a) and self-efficacy (RQ2b). As there is a great amount of evidence regarding the influence of supportive elements on learning performance and motivation, we expect that learning performance (H2a_recall_, H2a_comprehension_, and H2a_transfer_) and self-efficacy (H2b) are higher in the scaffolding groups than in the non-scaffolding groups.

The interaction of agency of regulation (system regulation/self-regulation) and scaffolding (with/without) on learning performance (RQ 3a) and self-efficacy (RQ 3b) cannot be predicted based on literature. Hence, we again investigate the interaction as an open research question (RQ3).

Finally, we analyze the influence of learner’s metacognitive competencies (RQ4a) and their prior knowledge (RQ4b) as moderators in the interplay of agency of regulation and scaffolding on learning performance. We expect a moderating effect of learner’s metacognitive competencies on learning performance (H4a_recall_, H4a_comprehension_, and H4a_transfer_) as well as a moderating effect of prior knowledge on learning performance (H4b_recall_, H4b_comprehension_, and H4b_transfer_).

## 2 Materials and methods

### 2.1 Sample and experimental design

The sample was *N* = 102 students of psychology and teacher education (75.5% female; 24.5% male) at a German University. Out of the original *N* = 112 students, *n* = 10 had to be excluded due to invalid data (lack of assignability of test subject codes). The average age of the participants was *M* = 20.97 (*SD* = 2.86) years and the current semester was *M* = 4.15 (*SD* = 2.649). To maintain ecological validity, a group of students of the educational science lecture in their realistic, natural learning setting was selected. As students increasingly have to deal with digital systems and self-regulated learning environments, the results can provide relevant insights into university contexts. Our 2 × 2 factorial between-subjects design included the factors agency of regulation (system regulation/self-regulation) and scaffolding (with/without). Within the design, learners received either a system-guided learning path with scaffolding, a system-guided learning path without scaffolding, a self-regulated learning path with scaffolding, or a self-regulated path without scaffolding. As dependent measures, we assessed learning performance (recall, comprehension, transfer), and self-efficacy. In addition, we included prior knowledge and metacognitive competencies as possible control variables to analyze the expected moderations. Randomization was conducted using the Unipark questionnaire software, whereby the students were randomly assigned to the four groups.

### 2.2 Learning environment and procedure

The learning material was provided in Moodle, a learning management system (LMS), and consisted of 8 thematic units (interactive H5P content including texts and pictures) on digitized teaching and learning. For each topic, the learners had to complete an intermediate test (including questions and tasks depending on the extent of the unit), which determined whether the learners could continue with the next learning content based on a percentage pass mark. In total, depending on the results of this intermediate test, the learning unit took about 1 h to complete. After completing the tests, the learners were given a hint about the further procedure in the scaffolding condition. Depending on the test result, the scaffolds differed according to success (e.g., “Well done! Based on your results, we recommend you now look at the chapter on learning strategies”) and failure (e.g., “Based on your test results, we recommend you to look at the material on e-teaching and flipped classroom again”). The groups without scaffolding only received the feedback about whether they passed or failed the respective test. The difference between the system-regulated and self-regulated groups was that the choice of learning content was mandatory in the system groups.

### 2.3 Measurement

The subjects had to complete an online questionnaire both before and after the learning unit. Domain-specific prior knowledge was assessed with 10 questions in the pretest. Besides 8 multiple-choice questions (e.g., “What are the phases of the metacognitive cycle?”), there was also a true/false task (e.g., “Rhythm distinguishes between the phases ‘information intake’ and ‘information output’”) and an open question (“What do you know about human memory?”). The question was rated by two independent coders, resulting in an interrater reliability of 0.73 *k*, which corresponds to substantial strength (Landis and Koch, 1977). A total of 16 points could be achieved in the prior knowledge test, with the questions weighted according to difficulty. A maximum of 3 points could be achieved in the open-ended task format, the true-or-false task was scored with 5 points (1 point per statement) and the multiple-choice tasks were weighted with 1 point each.

Learning performance was measured in the LMS by completing the tests at the end of each learning unit. The first attempt to complete a test was assessed as learning performance. Learners then had the opportunity to repeat the test until they passed it. Within the 8 test measurements, differentiation was made according to Bloom’s taxonomy (1956; recall, comprehension, and transfer). The distribution of tasks among the units depended on the complexity and length of the units. Across all units, at the recall level, 9 tasks were set (e.g., “Which phases take place in the flipped classroom at home?”). 10 tasks were aimed at comprehension (e.g., “What can you do to be cognitively active?”) and 6 tasks were aimed at transfer (“What are learning strategies? Alex cannot concentrate at all, does not feel like doing the tasks, and feels like he’s not taking much in. What do you advise Alex to do?”). The tasks were presented in formats such as drop-down lists, fill-in-the-blank, assessment of statements, or multiple choice. A maximum total of 45 points could be achieved by completing the 25 tasks to measure learning performance. Both prior knowledge and learning outcome measures covered a wide range of knowledge, which is why internal consistency is low compared to other psychological constructs and is not reported here.

To measure metacognitive competencies, we used the subscale on metacognition from the Learning Strategies in Study questionnaire (Wild and Schiefele, 1994) in the pretest, before the learners were asked to learn content on digitized teaching and learning. The 16 items cover both planning (e.g., I decide in advance how far I want to go in working through the material), monitoring (e.g., I ask myself questions about the material to make sure I have understood everything), and regulating (e.g., If a certain passage of text seems confused and unclear to me, I go through it again slowly) in the learning process. Internal consistency was a Cronbach’s alpha of *α* = 0.737. The 7-point Likert scale ranged from very rarely to very often. Demographic data such as age, gender, and semester were also collected. In addition, there were some evaluation items (self-generated) that asked about the appropriateness of the course on a scale from 1 (disagree at all) to 7 (agree completely; e.g., “I always knew what to do,” “The content of the course was coherent”).

From a motivational perspective, situation-related self-efficacy was recorded. The self-efficacy subscale was derived from the Motivated Strategies for Learning Questionnaire Manual MSLQ (Pintrich and DeGroot, 1990) and included 7 items (“e.g., “I currently expect to do well in this course”) that were intended to be rated on a scale of 1 (not at all true of me) to 7 (very true of me; *α* = 0.926).

### 2.4 Analysis methods

To test the hypothesis, ANOVAs or ANCOVAs were conducted using SPSS. We calculated moderated interactions with the independent factors agency of regulation and scaffolding on learning performance and the moderators’ metacognitive competencies and prior knowledge using the plugin PROCESS (model 3) by Hayes (2015). For analysis, we considered prior knowledge as a continuous variable. Alpha level *α* = 0.05 was used for all analyses. Effect sizes were interpreted based on Cohen (1988) work. For correlations, *r* = 0.10 is considered small, *r* = 0.30 is considered moderately strong, *r* = 0.50 is considered strong, and *r* = 0.80 is considered very strong (Cohen, 1988). For eta squared the following guidelines values were used: *η*^2^ = 0.01 corresponds to a small effect, *η*^2^ = 0.06 to a medium effect, and *η*^2^ = 0.14 to a large effect (Cohen, 1988).

## 3 Results

Descriptive analysis of demographic data illustrated that participants did not differ across groups regarding the pretest variables. We calculated one-factorial ANOVAs to ensure that there are no group differences in age (*p* = 0.874), prior knowledge (*p* = 0.725), and metacognitive competencies (*p* = 0.453). Gender also showed no significant group differences (*χ*^2^ (1, *N* = 102) = 4.25, *p* = 0.236). The descriptive data for the groups are presented in Table 2.

**Table 2:** Descriptive statistics including means and standard deviations.

Baseline characteristics	System regulation with scaffolding *n* = 21		System regulation without scaffolding *n* = 24		Self-regulation with scaffolding *n* = 32		Self-regulation without scaffolding *n* = 25		Full sample *N* = 102	
	*M*	*SD*	*M*	*SD*	*M*	*SD*	*M*	*SD*	*M*	*SD*
Pretest variables										
Age (years)	21.24	2.91	20.67	1.86	20.94	2.39	21.08	2.47	20.97	2.39
PK (%)	47.59	17.22	43.47	15.92	44.48	19.27	48.23	16.51	45.81	17.30
MC (1–7)	5.17	0.79	5.18	0.89	5.06	0.62	4.86	0.81	5.06	0.77
Posttest variables										
**Learning performance**										
Recall (%)	87.36	7.59	85.51	7.77	86.23	7.91	82.75	8.88	85.44	8.12
Comprehension (%)	79.24	9.42	78.17	7.40	81.72	9.40	78.41	13.31	79.57	10.08
Transfer (%)	63.39	11.98	60.03	9.87	61.46	11.01	65.00	13.88	62.39	11.71
SE (1–5)	4.85	1.13	4.51	1.03	4.82	1.12	4.32	1.38	4.63	1.17

PK, Prior knowledge; MC, Metacognitive Competencies; SE, Self-Efficacy.

In addition, we tested the potential relation of the control variables and the dependent variables using correlation analyses (see Table 3). This revealed that prior knowledge was significantly correlated with transfer (*r* = 0.40, *p* = <0.001). Accordingly, prior knowledge was included as a covariate in the calculation of ANCOVA for transfer. Metacognitive competencies were negatively correlated with learning performance on the recall level (*r* = −0.21, *p* = 0.038) and were consequently included in the respective analysis.

**Table 3:** Correlations for study variables.

Variable	1	2	3	4	5	6	7	8
Pretest variables								
1. Age	—							
2. Gender	−0.07	—						
3. PK	0.05	−0.08	—					
4. MC	0.01	−0.15	0.00	—				
Posttest variables								
**Learning performance**								
5. Recall	−0.17	−0.02	0.09	−0.21*	—			
6. Comprehension	0.07	−0.02	0.15	−0.17	0.40**	—		
7. Transfer	−0.01	−0.21^*^	0.40**	−0.04	0.04	0.15	—	
8. Self-efficacy	−0.05	0.07	0.11	0.11	0.22*	0.34**	0.33**	—

Pearson correlation matrix (*n* = 102). PK, Prior knowledge; MC, Metacognitive Competencies; ^*^p < 0.05. ^**^p < 0.01.

### 3.1 Effects of agency and scaffolds on learning outcomes and self-efficacy (RQ 1–3)

Regarding the first research question no effect of the agency of regulation on learning performance (RQ1a) was found at any performance level [recall: *F* (1, 97) = 2.614, *p* = 0.109, *η*_p_^2^ = 0.026; comprehension: *F* (1, 98) = 0.444, *p* = 0.507, *η*_p_^2^ = 0.005; transfer: *F* (1, 97) = 0.352, *p* = 0.554, *η*_p_^2^ = 0.004]. Concerning self-efficacy, no main effect of the agency of regulation (RQ1b) could be identified either [*F* (1, 98) = 0.298 *p* = 0.587, *η*_p_^2^ = 0.003].

The second research question (RQ2) focused on the main effects of scaffolding on learning performance and self-efficacy. Regarding learning performance, a significant but small main effect was found for recall [H2a_recall_: *F* (1, 97) = 3.733, *p_1_* = 0.028, *η*_p_^2^ = 0.037]. At the comprehension and transfer level, the ANOVA showed no main effects for scaffolding [H2a_comprehension_: *F* (1, 98) = 0.838, *p* = 0.362, *η*_p_^2^ = 0.008; H2a_transfer_: *F* (1, 97) = 0.009, *p* = 0.924, *η*_p_^2^ = 0.000]. However, a significant but small effect of scaffolding on self-efficacy could be demonstrated [H2b: *F* (1, 98) = 4.092, *p_1_* = 0.046, *η*_p_^2^ = 0.040].

Regarding our third research question (RQ3), the interaction of both factors, no effects were revealed for neither level of learning performance [recall: *F* (1, 97) = 0.592, *p* = 0.443, *η*_p_^2^ = 0.006; comprehension: *F* (1, 98) = 0.156, *p* = 0.694, *η*_p_^2^ = 0.002; transfer: *F* (1, 97) = 0.876, *p* = 0.352, *η*_p_^2^ = 0.009] and also not for self-efficacy [*F* (1, 98) = 0.330, *p* = 0.567, *η*_p_^2^ = 0.003].

### 3.2 Moderating effects of metacognitive competencies and prior knowledge (RQ 4)

The assumption, that the metacognitive competencies moderate the interaction of the agency of regulation and scaffolding on learner performance (H4a), could not be confirmed. The overall models and the individual interactions were insignificant for each learning level [H4a_recall_: *R*^2^ = 10,67, *F* (7, 94) = 1.603, *p* = 0.144; H4a_comprehension_: *R*^2^ = 9,17, *F* (7, 94) = 1.355, *p* = 0.234; H4a: *R*^2^ = 2,02, *F* (7, 94) = 0.276, *p* = 0.962].

**Figure 1:**
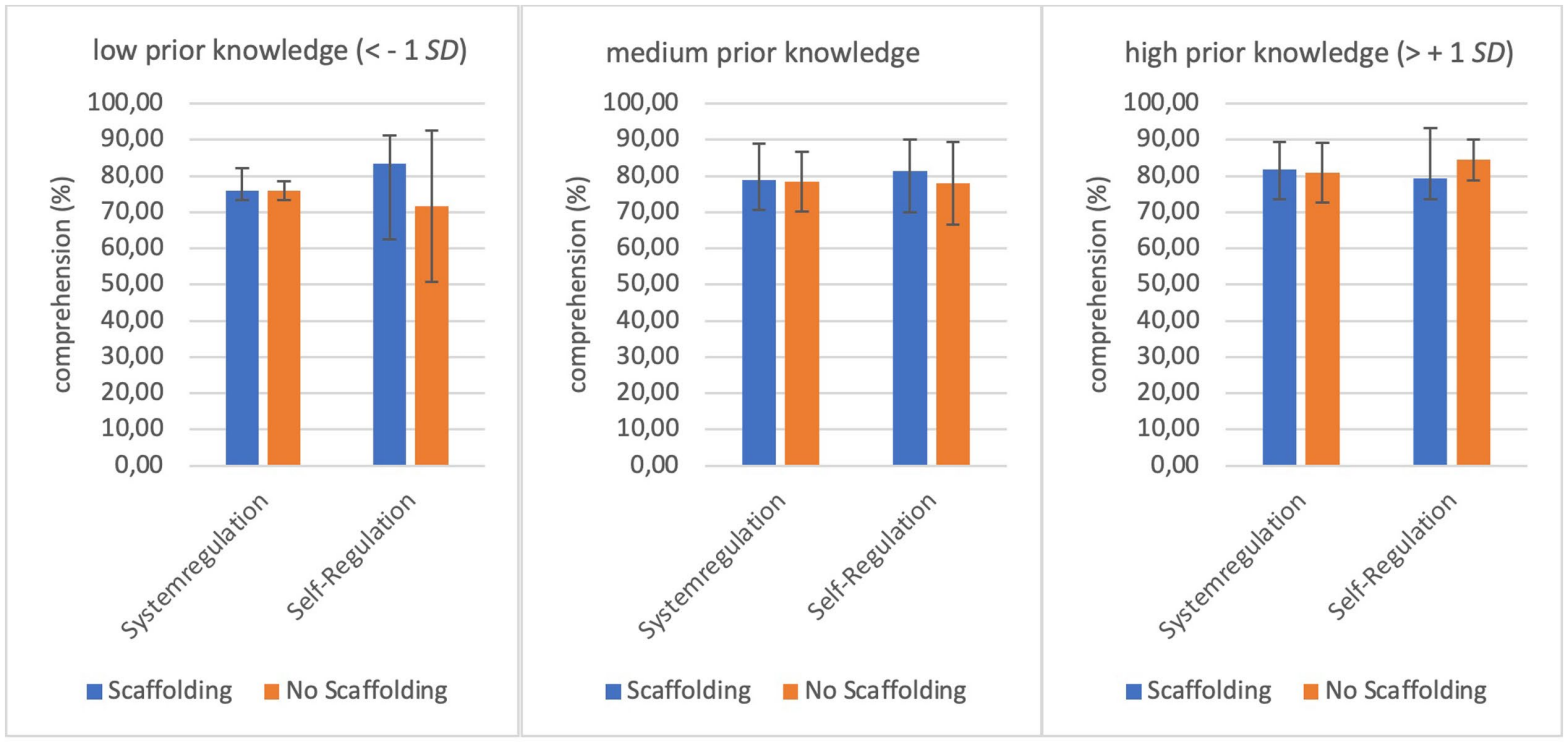
Means and standard deviations of comprehension depending on the different conditions for low, medium, and high prior knowledge.

Regarding prior knowledge as a moderator for the interaction between the agency of regulation and scaffolding on learning performance, we found no significant results for recall (H4b_recall_) and transfer (H4b_transfer_). Concerning recall, we did not find a significant overall model [H4b_recall_: *R*^2^ = 8,02, *F* (7, 94) = 1.170, *p* = 0.327]. Even if the overall model for transfer was significant [H4b_transfer_: *R*^2^ = 21,93, *F* (7, 94) = 3.773, *p* = 0.001], the individual interactions were not. The hypotheses H4b_recall_ and H4b_transfer_ must therefore be rejected. Concerning comprehension (H4b_comprehension_), however, a moderated interaction between the agency of regulation, scaffolding, and prior knowledge could be demonstrated. In addition to a significant overall model [H4b_comprehension_: *R*^2^ = 14,27, *F* (7, 94) = 2.236, *p* = 0.038], the interactions between the type of regulation and scaffolding [*b* = 26.20, *t*(94) = 2.29, *p* = 0.02] as well as the interaction between regulation, scaffolding, comprehension, and prior knowledge were significant {*R^2^* = 4,36, *F* (1, 94) = 4.781, *p* = 0.031, 95% CI [−6.101, −0.294]}. The model has a moderate variance resolution of 14.27% (Cohen, 1988). Figure 1 visualizes the means and standard deviations of comprehension, depending on the different conditions for the fictional groups with low (1 *SD* lower than the mean), medium (mean), and high prior knowledge (1 *SD* above mean).

## 4 Discussion

The objective of the study was to investigate the effect of the agency of regulation and scaffolding on learning performance and self-efficacy. In addition, the learners’ metacognitive competencies and prior knowledge were considered as potential moderators.

### 4.1 Effects of agency and scaffolds on learning performance

Concerning learning performance, different interpretations can be derived from the results. Firstly, there was no main effect for the different levels of learning performance (RQ1a). Based on our theoretical preliminary work, we expected that neither system nor self-regulation is generally more conducive to learning, which possibly depends on further complex factors (e.g., sense of autonomy, extend of learner control, self-regulatory strategies). On one site, the learning-promoting effect of system regulation was perhaps undermined by the restriction on self-regulation. Consequently, learners had no opportunity to regulate themselves (e.g., set their own goals, choose appropriate learning strategies, and monitor their decision-making) which is why they learned less effectively (Shirk, 2020). This assumption comes up frequently when internal and external regulations are contrasted (Guo, 2022; Kerres and Buntins, 2020). Conversely, the effect of self-regulation on learning performance may have been limited by the fact that learners did not have enough control to set their own goals or used ineffective strategies (Azevedo et al., 2008).

Moreover, especially for the learners in our learning setting, it may not have mattered fundamentally whether the system or the learners themselves regulated. It is conceivable that the system regulation was not experienced as such by the learners, as the students are used to the linear structure in the LMS and to predefined learning paths. However, learners who self-regulated their learning may also have been used to finding their way around the LMS and organizing their learning themselves, as they usually do in other courses. The main difference between the two conditions, apart from the control to regulate learning, was the visibility of the subsequent learning content, which consequently had no significant impact on learning.

In terms of the scaffolding factor, a significant main effect was found for recall (H2a_recall_). Simultaneously, scaffolding had no significant effect on comprehension (H2a_comprehension_) and transfer (H2a_transfer_). In principle, we were able to replicate that scaffolding can increase learning performance (Azevedo et al., 2022; Guo, 2022), but only the recall of learning content. A testing effect may have occurred (Johnson and Mayer, 2009; Malmberg et al., 2014). Thus, due to the scaffolding, the learners recognized the relevance of the learning performance tests in the course of the learning session and learned better accordingly, which is presumably why recall increased. Learners were therefore more likely to recall the knowledge as they remembered the content through the tests. At the same time, they may have adapted their knowledge retrieval strategies after realizing that the tests were recurring (Malmberg et al., 2014). However, the scaffolds only increased recall. Although the learning performance test assessed recall, comprehension, and transfer, scaffolding did not result in deeper cognitive processing. We must therefore ask ourselves why the learners did not also engage at the higher levels according to Bloom et al. (1956) assumptions. It is plausible, that the scaffolds were quite simple and only informed the learner about their test results and further procedures. In-depth processing might therefore have had to be specifically prompted through the scaffolds to improve comprehension and transfer. Bannert et al. (2009) suggested a concrete approach to support deeper processing. By indicating to learners that they must subsequently be able to explain what they have learned to others, comprehension and transfer can be promoted (Bannert et al., 2009). Another explanation can be found in the difficulty and didactic preparation of the learning material. Although the learners had an average prior knowledge, there was a higher percentage of learning performance at recall, compared to the upper levels. It is feasible that the learning content did not prepare the learners to answer more difficult tasks for comprehension and transfer, which is why scaffolding only affected recall. This could be counteracted by enriching the material with more practical and instructional examples (Van Gog et al., 2004).

There was no interaction of both factors on recall, comprehension, and transfer (RQ3). Scaffolding in the system condition aimed to create transparency about the decision-making process of the system and thus increase learning performance. At the same time, in the self-regulation condition, scaffolding was intended to provide metacognitive support to help learners reflect on their learning process and thereby improve learning. We can therefore conclude that there is no interplay between the agency of regulation and scaffolding on learning performance and further individual criterions (e.g., prior knowledge, feeling of autonomy) should be taken into consideration.

### 4.2 Effects of agency and scaffolds on self-efficacy

Concerning self-efficacy, no effect of the agency of regulation (RQ1b), but an effect of scaffolding (RQ2b) was found. Self-efficacy therefore did not differ in the regulation groups. One potential explanation for this is that self-efficacy is relatively static compared to other motivational states. Depending on how it is measured, self-efficacy can be understood as a personality trait or motivational state. To capture the situational change in self-efficacy, a sensitive and repeated process measurement would have been more accurate (McQuiggan et al., 2008). The agency of regulation might then have had a more measurable impact. This assumption is contradicted by the fact that scaffolding had a main effect on self-efficacy. Consequently, the measurement in this case was sensitive enough to detect a difference. We conclude that self-efficacy is a relatively stable, but nevertheless influenceable construct.

Moreover, the fact that instructional aids such as scaffolding have an impact on self-efficacy is consistent with other recent research (Guo et al., 2023; Valencia-Vallejo et al., 2018). The supporting intervention may encourage the learner to reflect on their learning process. This in turn increases their belief in their abilities and their capacity to achieve learning goals through their efforts (Valencia-Vallejo et al., 2018). About our learning scenario, we can draw the following conclusion. The scaffolding encouraged learners to believe that they have control over the successful application of their acquired knowledge in a test. In this way, the repeated scaffolding signalized their progress in learning, whereby the learner experienced higher self-efficacy.

### 4.3 Moderating effects of metacognitive competencies and prior knowledge

To test our research question (RQ4), we examined the moderator variables metacognitive competencies and prior knowledge. Contrary to our expectation, no moderation by the learner’s metacognitive competencies could be demonstrated (H4a). One possible explanation for this could have been the type of measurement. Most of the items used are related to study learning in general and not to digital learning. However, a more specific assessment of metacognitive competencies would have provided additional insight into individual self-regulatory strategies and skills. This would have allowed a more detailed analysis of metacognitive competencies as a moderator.

In general, the participants had a rather high level of metacognitive competencies. However, we presumed that learners with low metacognitive competencies can benefit from the regulation by the system and the scaffolding concerning their learning outcome, as they are relieved in making learning decisions (Kerres and Buntins, 2020). We also assumed that in self-regulated learning settings, learners with low metacognitive competencies can benefit from scaffolding, as self-regulatory deficits are compensated (Bannert, 2009). Consequently, the sample may not have been heterogeneous enough to confirm these assumptions.

The moderation by prior knowledge (H4b) was not significant for recall and transfer, but regarding comprehension. Depending on the learners’ prior knowledge, it makes a difference for comprehension whether the system or the learners themselves regulated and whether they received scaffolding. It made no difference in the system regulation condition for learners with low prior knowledge, whether they were supported with scaffolding or not regarding comprehension. This result does not meet our expectations and is possible because the learners did not yet have enough prior knowledge to use the instructional aid appropriately (Seufert, 2003). As expected, scaffolding had a larger impact on comprehension than no scaffolding for learners with low prior knowledge in the self-regulation condition. For learners with medium prior knowledge, scaffolding also had a more beneficial effect on comprehension than no scaffolding, although the difference is more pronounced in the self-regulation condition compared to the system regulation condition. This is consistent with the assumption that higher prior knowledge is associated with the ability to cope with a self-regulated situation (Greene et al., 2010). For learners with a high level of prior knowledge, scaffolding only had a supportive effect on comprehension in the system regulation condition. In the self-regulation condition, learners no longer needed support to learn better. This picture replicates the expertise reversal effect (Kalyuga et al., 2003). Learners who already had a high level of prior knowledge no longer benefit from the support. This is also shown by previous research (Seufert, 2003). The exact cause of this phenomenon remains unclear. It is possible that the learners perceived the help as disruptive (Roelle and Berthold, 2013) or simply no longer needed it (Seufert, 2003). However, the question arises as to why the scaffolds in the system-regulated condition still had a supportive effect on comprehension even for learners with a high level of prior knowledge. One possible explanation would be that the learners were prevented from regulating themselves, as the system took responsibility for this (Kerres and Buntins, 2020). Consequently, learners with high prior knowledge still profited from the scaffolding. Moreover, it is feasible that in system-regulated contexts, the scaffolding supported the learner to comprehend the system’s decisions and gain an advantage in their learning (Vandewaetere and Clarebout, 2011).

Finally, we also have to ask why the moderating effect of prior knowledge was not found on recall and transfer. Bloom’s taxonomy (1956) assumes in principle that learners must first be able to recall and comprehend what they have learned before they can transfer it to other contexts. The percent learning performance in our sample mirrors this hierarchy. Learners had the highest learning outcome in terms of recall (*M* = 85.44%), followed by comprehension (*M* = 79.57%) and transfer (*M* = 62.39%). In principle, we would therefore expect prior knowledge to become more relevant with increasing complexity. However, it is also conceivable that prior knowledge is less relevant, as recall is quite superficial, while during transfer learned is already internalized. Nevertheless, it is also likely that the measurement of learning performance in our study was not sufficiently broad enough to detect further differences. Comprehension involves recognizing connections and structures in learning material, differentiating it from recall. The structure-giving scaffolding could therefore be a further explanation, as it assisted the learner to better understand the logical patterns and connections in the overall structure of the learning topic (Zheng, 2016).

### 4.4 Limitations and future research

Some limitations should be taken into account when classifying this study. The goal was to create a natural learning environment and offer learners maximum freedom to draw relevant conclusions in a university setting. To improve the generalizability of the results, the study should be repeated on a sample from a different domain or a larger domain-independent sample. In addition, the learning unit of the experiment was embedded in a course, which is why the sample size was limited from the outset. Technical restrictions arose due to the linking of conditions when creating learning paths, which further increased the dropout rate.

Another limitation is the differentiation of agency conditions. With regard to the learning unit and the LMS, it should be emphasized that the system regulation was simulated with hard-wired learning paths, which may limit the authenticity of the system. In this context, it is also important to note that the variation in system-regulated conditions was marginal. The learners with and without scaffolding could only decide for themselves whether they would repeat learning content. In the case of scaffolding, the aim of the hint was to create transparency regarding the system’s decision. But despite the rather small difference in the instruction, the positive effect on recall was significant.

Shute and Zapata-Rivera (2012) point out that obtaining accurate data about learners as a foundation for adaptive decisions represents a major barrier to progress in the field of adaptive educational technologies. Using the simulated system, we were able to overcome privacy concerns and establish a direct comparison between agency conditions. With regard to self-regulation in the learning unit, it should be noted that although the learners were able to determine the order of the content themselves, the setting and content were predetermined and possibly did not require self-regulation to this extent. Therefore, when designing learning environments, it is important to provide learners with the right amount of self-regulation and control by the system without controlling them too drastically (Azevedo et al., 2008; Kalyuga, 2009; Shirk, 2020). In the context of complex learning processes, research into shared learner control requires further fine-grained studies that incorporate both the dynamic nature of self-regulated learning and the rapid evolution of learning systems.

Moreover, the learners could repeat the tests we used for measuring learning performance. They might have used this as a learning strategy. Only the first attempt was recorded as a learning success. Nevertheless, a testing effect could have arisen here (Malmberg et al., 2014). Table 3 also shows that the learning outcome variables are hardly correlated with each other. This is plausible with regard to the lower levels, as learners can recall content but do not have to comprehend it at the same time. However, the transfer tasks may have been too abstract or too far from the knowledge base.

Although the potential of scaffolding was demonstrated in this study, future studies should investigate adaptive scaffolding (e.g., through fading guidance; Guo, 2022; Lim et al., 2023; Van Merriënboer and Sluijsmans, 2009; Zheng, 2016). Especially in combination with the regulatory agency, an interaction effect on learning performance may be more likely to be proven, as adaptive scaffolding is a tailored support measure. In addition to cognitive measurements, such as learning performance, a more precise recording and investigation of metacognitive and motivational (e.g., autonomy) processes during learning could provide more information about individual needs. The recording of self-regulation deficits during learning would make it possible to intervene adaptively with a scaffold or an adapted degree of system regulation. At the same time, measuring motivational states could be used to respond to individual motivation lows. However, it should be taken into account that the self-reported measurement of self-regulation in particular is controversial (Panadero et al., 2016). To make targeted adjustments, a precise analysis of the learning process is required. Thus, peripheral data (e.g., mouse or typing behavior) should also be included in the decision for individual support of self-regulation (Hörmann and Bannert, 2016).

## 5 Conclusion

In summary, our study has demonstrated that scaffolding promotes both learners’ recall of knowledge and self-efficacy. In practice, this indicates that teachers and educational system developers can provide targeted support in the design of didactic materials using instructional aids. Although the results suggest that even generalized scaffolding has a supportive influence, individual scaffolding could lead to better results (Guo, 2022; Schwonke et al., 2006; Zheng, 2016).

Regarding the agency of regulation, it remains a central challenge to obtain a suitable balance between system and self-regulation for each learner. While system regulation has the potential to support the learner individually, to improve self-regulatory skills and thus learning performance (Harati et al., 2020; Shute and Zapata-Rivera, 2012; Tetzlaff et al., 2021; Vandewaetere and Clarebout, 2011), self-regulation offers the benefit that the learner’s control supports the learner in managing a learning task and thereby controlling motivational, emotional, cognitive and metacognitive characteristics of learning. Learners are thus enabled to learn more effectively in the long term (Panadero, 2017). This implies that neither system nor self-regulation is the one solution for each learner. Depending on the individual requirements of the learner and the respective learning situation, the learner or the system should be in control of the learning process. However, extensive studies are still required to draw clear recommendations.

Finally, we were able to show that together, the agency of regulation and scaffolding increases learners’ comprehension depending on their prior knowledge, which is why individual prior knowledge should be taken into account when designing learning paths and scaffolding to enhance learning performance (comprehension).

In conclusion, it should be mentioned that future studies need to examine supporting interventions in the context of system- and self-regulated learning in greater depth to identify further insights. This could be achieved through detailed and dynamic analyses of the learning process (e.g., goal setting, monitoring, and strategy use) in both system-based and self-regulated learning. One approach to do so is the inclusion of prior knowledge as a basis for adaptation and the measurement of individual self-regulatory processes to provide adaptive scaffolds.

## Data Availability

The raw data supporting the conclusions of this article will be made available by the authors, without undue reservation.
