# Corrigendum: Who regulates? Effects of scaffolding in system- and self-regulated learning

**DOI:** 10.3389/fpsyg.2025.1642170

**Published:** 2025-06-23

**Authors:** Aileen Herold, Tina Seufert

**Affiliations:** Department Learning and Instruction, Institute of Psychology and Education, Faculty of Engineering, Computer Science and Psychology, University of Ulm, Ulm, Germany

**Keywords:** learning systems, self-regulation, scaffolding, learning performance, self-efficacy, prior knowledge

In the published article, there was a mistake in Table 2 as published. The table lists a variable that should not be included [PK (max = 16)]. The correct variable has already been inserted under PK (%).

The corrected Table 2 appears below.

**Table 2:** Descriptive statistics including means and standard deviations.

**Baseline characteristics**	**System regulation with scaffolding** ***n*** = **21**		**System regulation without scaffolding** ***n*** = **24**		**Self-regulation with scaffolding** ***n*** = **32**		**Self-regulation without scaffolding** ***n*** = **25**		**Full sample** ***N*** = **102**	
	***M***	***SD***	***M***	***SD***	***M***	***SD***	***M***	***SD***	***M***	***SD***
**Pretest variables**										
Age (years)	21.24	2.91	20.67	1.86	20.94	2.39	21.08	2.47	20.97	2.39
PK (%)	47.59	17.22	43.47	15.92	44.48	19.27	48.23	16.51	45.81	17.30
MC (1–7)	5.17	0.79	5.18	0.89	5.06	0.62	4.86	0.81	5.06	0.77
**Posttest variables**										
**Learning performance**										
Recall (%)	87.36	7.59	85.51	7.77	86.23	7.91	82.75	8.88	85.44	8.12
Comprehension (%)	79.24	9.42	78.17	7.40	81.72	9.40	78.41	13.31	79.57	10.08
Transfer (%)	63.39	11.98	60.03	9.87	61.46	11.01	65.00	13.88	62.39	11.71
SE (1-5)	4.85	1.13	4.51	1.03	4.82	1.12	4.32	1.38	4.63	1.17

PK, prior knowledge; MC, metacognitive competencies; SE, self-efficacy.

The authors apologize for this error and state that they do not change the scientific conclusions of the article in any way. The original article has been updated.

